# Changes in Antimicrobial Usage Patterns in Korea: 12-Year Analysis Based on Database of the National Health Insurance Service-National Sample Cohort

**DOI:** 10.1038/s41598-018-30673-6

**Published:** 2018-08-15

**Authors:** Young Ah Kim, Yoon Soo Park, Taemi Youk, Hyukmin Lee, Kyungwon Lee

**Affiliations:** 1 Department of Laboratory Medicine, National Health Insurance Service Ilsan Hospital, Goyang, Korea; 2 Department of Internal Medicine, National Health Insurance Service Ilsan Hospital, Goyang, Korea; 3 Research Institute, National Health Insurance Service Ilsan Hospital, Goyang, Korea; 4 Department of Laboratory Medicine and Research Institute of Bacterial Resistance, Yonsei University College of Medicine, Seoul, Korea

## Abstract

National antimicrobial usage and prescription patterns during the 12 years from 2002 to 2013 were analyzed using the National Health Insurance Service-National Sample Cohort. Antimicrobial usage was analyzed by major illness, sex, age, area of residence, income rank, diagnosis, and type of medical institution for each year. Total antimicrobial prescriptions increased from 15.943 daily defined dose (DDD)/1,000 inhabitants/day in 2002 to 24.219 in 2013. In 2013, 72% of total prescriptions were administered in clinics. Antimicrobials were most frequently prescribed to children younger than 10 years, followed by adults aged 70 years or older and those aged 60–69 years. Penicillins and cephems were the most popular classes of antimicrobial used. In 2013, 48% of total antibiotic usage (11.683 DDD/1,000 inhabitants/day) was due to respiratory diseases. After the Korean government has implemented a series of healthcare policies, antibiotic prescription decreased for the treatment of upper respiratory infection, the causative agents are mostly viruses.

## Introduction

Although antibiotics have saved the lives of millions of people worldwide since the discovery of penicillin in 1928, the emergence of antibiotic resistance in bacteria is a serious global challenge. Antibiotic resistance develops when bacteria adapt and grow in the presence of antibiotics. Because the development of resistance is linked to how often antibiotics are used, misuse and over-use of antibiotics hasten the development of bacterial drug resistance, rendering existing antibiotics less effective^1–3^. Although infection by multidrug-resistant bacteria poses a grave threat to public health and wellbeing through subsequent increases in hospital stays, medical costs, and mortality^4–6^, the development of new antimicrobials is a practically challenging task.

Because bacterial resistance is increasing and the development of new antimicrobial is declining, securing the efficacy of existing antibiotics for long periods of time is the key to solving the antimicrobial resistance problem. In other words, the key is to suppress the occurrence of bacterial resistance in existing antibiotics. In 2015, World Health Organization (WHO) adopted a global action plan on antimicrobial resistance, which declared it as an urgent public health concern and emphasized prudent antimicrobial prescriptions in human and animal health, based on evidence and the current epidemiology^1^.

Although the prevalence of antimicrobial resistance in Korea is higher than that in advanced countries such as the United States and Europe^7,8^, studies on the changes in antimicrobial usage patterns, which drive antimicrobial management policies, were limited in terms of research institutions, regions, and medical facilities^9–16^. The aim of this study was to analyze antimicrobial usage and prescription patterns at the national level in Korea during the 12-year period from 2002 to 2013. We expect the findings to help identify the major groups in need of intervention and provide grounds for developing public health policies to manage antimicrobial usage.

## Methods

### Analysis of antimicrobial usage and prescription patterns

We analyzed national antimicrobial usage and prescription patterns during the 12 years from 2002 to 2013 using the National Health Insurance Service-National Sample Cohort (NHIS-NSC)^17^. The data obtained from the national health database consisted of a national sample of 1,025,340 people (males: 513,258, females: 512,082), accounting for 2.2% of the total Korean population as of the 2002 census.

Systematic stratified sampling was used based on sex, age, and type of health insurance, and the data include socioeconomic variables (e.g., area of residence, income rank, disability), mortality, healthcare use (e.g., medical bills, details of care, diagnosis, and prescription), and health examination data.

Antimicrobials were classified as penicillins, cephems, carbapenems, fluoroquinolones, aminoglycosides, tetracyclines, macrolides, glycopeptides, or folate path inhibitors for analysis. Antimicrobial usage was analyzed by income rank (5 ranks), which was determined using insurance bills per household, by medical institution (general hospital, hospital, long-term care hospital, clinic), and by region (17 cities and provinces). Following medical law in Korea, we classified medical institutions as clinics if they had fewer than 30 beds, as hospitals if they had more than 30 beds, and as general hospitals if they had more than 100 beds. A long-term care hospital was defined as a nursing hospital if it was intended for patients with geriatric or chronic diseases or patients who require postoperative recovery.

Upper respiratory infection (URI) was included as a major illness for analysis of antimicrobial usage patterns. URI, which involves viral pathogens in most cases, rarely requires antimicrobials for treatment and is associated with antimicrobial abuse. Bacterial pneumonia and urinary tract infections require antimicrobial prescriptions, and the incidence of these diseases is increasing with the growing elderly population. The major illnesses analyzed in this study, according to the Korean Standard Classification of Diseases, were respiratory diseases (code J) and genitourinary diseases (code N); more specifically, we analyzed URIs (J00-J06), pneumonia (J13-J17), and urinary tract infections (N10-N12, N136, N151, N159, N20-N21, N30, N34, N390).

Because the dose, form, and frequency of use vary across antimicrobial products, the daily defined dose (DDD) and yearly antimicrobial usage by active ingredient were calculated as follows to measure standardized usage.

$$\begin{array}{c}{\rm{Antimicrobial}}\,{\rm{usage}}\,{\rm{by}}\,{\rm{active}}\,{\rm{ingredient}}\,({\rm{DDD}}/1,\,000\,{\rm{inhabitants}}/{\rm{day}})\\ \,=\frac{{\rm{Amount}}\,{\rm{of}}\,{\rm{antimicrobials}}\,{\rm{used}}\,{\rm{for}}\,{\rm{a}}\,{\rm{year}}\,({\rm{mg}})}{{\rm{WHO}}-{\rm{DDD}}\,({\rm{mg}})\times 365\,days\times {\rm{sample}}\,\mathrm{population}\,\,{\rm{of}}\,{\rm{the}}\,{\rm{year}}}\,\,\,\,\times 1,\,000\,{\rm{inhabitants}}\end{array}$$

This describes the number of people (per a population of 1,000) who use an antimicrobial every day. As an example, 10 DDD per 1,000 inhabitants per day means that 1% of the population, on average, might receive a certain drug or group of drugs daily. Antimicrobial usage was analyzed by sex, age, area of residence, income rank, diagnosis, and type of medical institution for each year.

### Statistical analysis

Trend tests were performed to determine potential differences in antimicrobial usage by year, and linear mixed models using group, time, and group-by-time as fixed effects were used to identify potential differences in the yearly patterns of antimicrobial usage in relation to general characteristics. Data were organized and analyzed using SAS v9.4 software (SAS Institute Inc., Cary, NC, USA).

## Results

### Antimicrobial prescriptions

**Figure 1:**
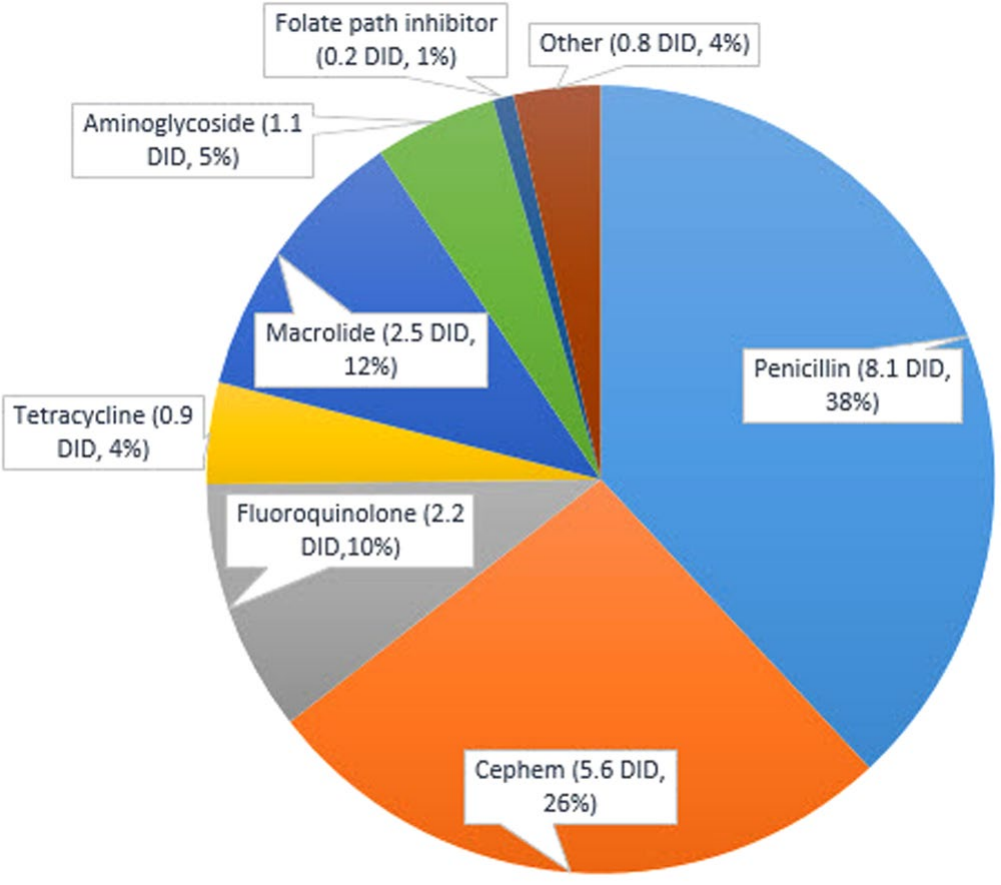
Average antimicrobial prescription according to class of antimicrobials from 2002 to 2013 (DID: Daily Defined Dose/1,000 inhabitants/days).

Total antimicrobial prescriptions in Korea increased from 15.943 DDD/1,000 inhabitants/day in 2002 to 24.219 in 2013 (Table 1, *P* < 0.001). By class, penicillins were the most frequently prescribed, followed by cephems, including cephalosporin and cephamycin (Fig. 1). No changes in penicillin prescription patterns occurred over the course of the 12-year period (*P* = 0.661). Cephem, carbapenem, fluoroquinolone, tetracycline, macrolide, and glycopeptide prescriptions increased, and aminoglycoside and folate path inhibitor prescriptions decreased (Table 1, *P* < 0.001).

**Table 1:** Antimicrobial prescription according to class of antimicrobials (DDD/1,000 inhabitants/days).

Class	2002	2003	2004	2005	2006	2007	2008	2009	2010	2011	2012	2013	*P*
Penicillin	7.654 (48)	7.228 (44)	8.207 (46)	8.868 (46)	8.484 (42)	7.796 (38)	8.640 (36)	8.273 (34)	8.527 (33)	7.990 (32)	8.008 (32)	7.768 (32)	0.661
Cephem	3.245 (20)	3.535 (21)	3.883 (21)	4.385 (22)	4.930 (24)	5.343 (26)	6.575 (27)	6.723 (28)	7.241 (28)	7.134 (28)	7.327 (29)	7.211 (29)	<0.001
Carbapenem	0.003 (0)	0.008 (0)	0.013 (0)	0.016 (0)	0.024 (0)	0.029 (0)	0.036 (0)	0.033 (0)	0.041 (0)	0.045 (0)	0.051 (0)	0.055 (0)	<0.001
Fluoroquinolone	1.445 (9)	1.665 (10)	1.730 (9)	1.891 (9)	1.953 (9)	2.225 (10)	2.630 (10)	2.567 (10)	2.578 (10)	2.614 (10)	2.669 (10)	2.565 (10)	<0.001
Tetracycline	0.406 (2)	0.427 (2)	0.434 (2)	0.459 (2)	0.581 (2)	0.857 (4)	1.102 (4)	1.161 (4)	1.270 (5)	1.301 (5)	1.336 (5)	1.334 (5)	<0.001
Macrolide	0.999 (6)	1.263 (7)	1.440 (8)	1.724 (8)	1.970 (9)	2.300 (11)	2.850 (11)	3.067 (12)	3.450 (13)	3.627 (14)	3.583 (14)	3.392 (14)	<0.001
Glycopeptide	0.006 (0)	0.011 (0)	0.014 (0)	0.015 (0)	0.020 (0)	0.029 (0)	0.038 (0)	0.034 (0)	0.039 (0)	0.043 (0)	0.047 (0)	0.053 (0)	<0.001
Aminoglycoside	1.494 (9)	1.266 (7)	1.204 (6)	1.166 (6)	1.062 (5)	1.047 (5)	1.049 (4)	1.017 (4)	0.973 (3)	0.951 (3)	0.838 (3)	0.807 (3)	<0.001
Folate path inhibitor	0.231 (1)	0.249 (1)	0.218 (1)	0.201 (1)	0.182 (0)	0.176 (0)	0.179 (0)	0.155 (0)	0.155 (0)	0.150 (0)	0.145 (0)	0.140 (0)	<0.001
Total amount	15.943	16.142	17.656	19.272	19.753	20.482	23.958	23.836	25.107	24.732	24.899	24.219	<0.001

All classes of antimicrobial were included in total amount. (% of total DDD).

Data from 2013 were examined to identify the antimicrobials most frequently prescribed in recent years. The total penicillin prescription rate was 7.768 DDD/1,000 inhabitants/day, and antimicrobials containing amoxicillin were the most frequently used. Amoxicillin was popular in 2002, but amoxicillin-clavulanate was more frequently used by 2013 (in DDD/1,000 inhabitants/day: amoxicillin 5.214, amoxicillin-clavulanate 1.777 in 2002; amoxicillin 2.250, amoxicillin-clavulanate 4.943, amoxicillin-sulbactam 0.394 in 2013). Among the cephems, cefaclor (3.222) was the most frequently prescribed, followed by cefuroxime (0.837), and cephradine (0.438). These three drugs accounted for 61% of all cephem prescriptions (7.211). Among fluoroquinolones, levofloxacin (0.978) was the most frequently prescribed, followed by ciprofloxacin (0.675), and ofloxacin (0.594). These three drugs accounted for 89% of the total fluoroquinolone prescriptions (2.565). The total macrolide prescriptions equaled 3.392 DDD/1,000 inhabitants/day, consisting of clarithromycin (1.896), roxithromycin (1.301), and azithromycin (0.193). For tetracycline, aminoglycoside, folate path inhibitor, carbapenem, and glycopeptide, 1.334, 0.807, 0.14, 0.055, and 0.053 DDD/1,000 inhabitants/day were prescribed, respectively.

**Figure 2:**
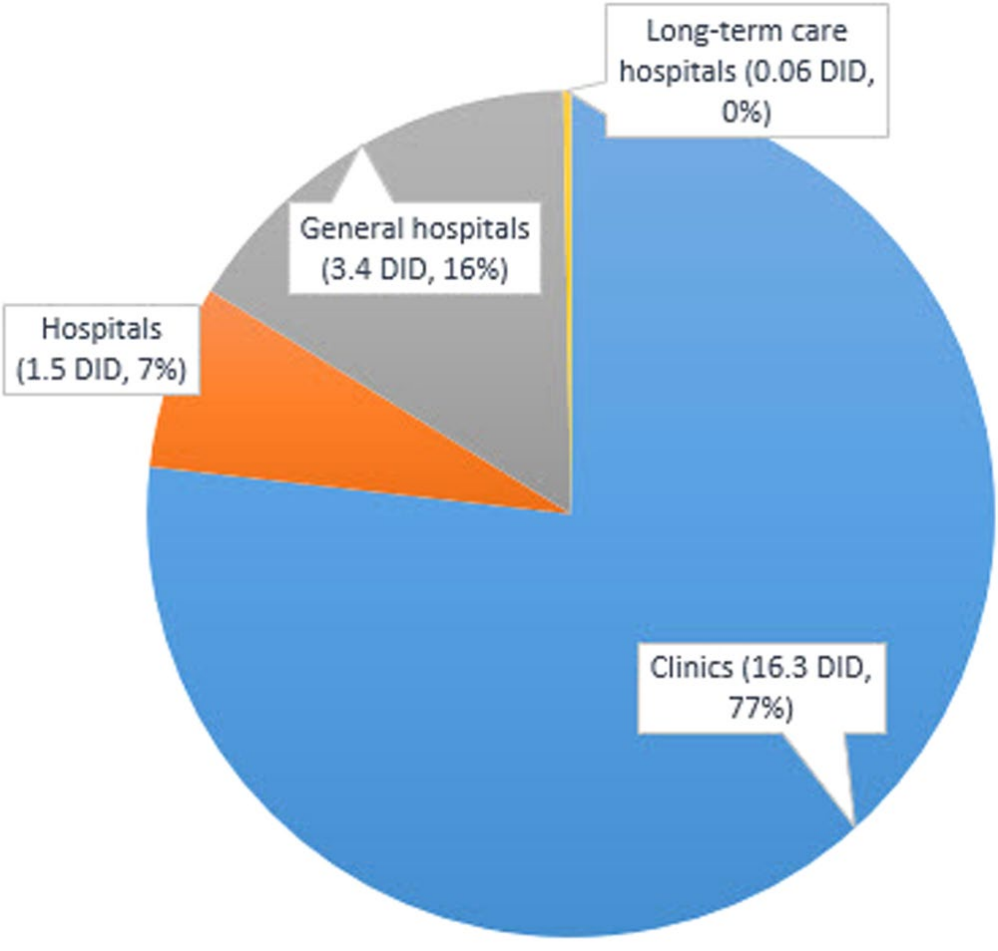
Average antimicrobial prescription according to types of medical institutions from 2002 to 2013 (DID: Daily Defined Dose/1,000 inhabitants/days).

Regarding the types of medical institutions, the vast majority of antimicrobials were prescribed in clinics, followed by general hospitals, hospitals, and long-term care hospitals (Fig. 2). In 2013, 72% of total prescriptions occurred in clinics (Table 2). Among clinics, penicillins (6.378 DDD/1,000 inhabitants/day) were the most frequently prescribed antimicrobial, followed by cephems (4.796), macrolides (2.639), and fluoroquinolones (1.536). In general hospitals, cephems (1.443) were the most frequently prescribed antimicrobial, followed by fluoroquinolones (0.767), penicillins (0.635), and macrolides (0.488) (Table 2). Antimicrobial prescriptions of all classes, except for that of penicillin in clinics (*P* = 0.111), increased every year regardless of medical institution (Table 2).

**Table 2:** Antimicrobial prescription according to medical institution (DDD/1,000 inhabitants/days). All classes of antimicrobial were included in total amount. (% of subtotal DDD or % of total DDD).

Medical institution	Class	2002	2003	2004	2005	2006	2007	2008	2009	2010	2011	2012	2013	*P*
Clinics	Penicillin	7.252 (51)	6.579 (49)	7.367 (52)	7.839 (52)	7.331 (49)	6.725 (43)	7.467 (40)	7.045 (38)	7.242 (38)	6.683 (37)	6.650 (36)	6.378 (36)	0.111
	Cephem	2.735 (19)	2.692 (20)	2.805 (19)	3.035 (20)	3.047 (20)	3.625 (23)	4.628 (25)	4.783 (26)	5.078 (26)	4.769 (26)	4.919 (27)	4.796 (27)	<0.001
	Fluoroquinolone	1.154 (8)	1.260 (9)	1.277 (9)	1.357 (9)	1.384 (9)	1.573 (10)	1.879 (10)	1.759 (9)	1.678 (8)	1.628 (9)	1.644 (9)	1.536 (8)	0.006
	Macrolide	0.796 (5)	0.913 (6)	1.028 (7)	1.235 (8)	1.415 (9)	1.700 (10)	2.191 (10)	2.374 (13)	2.679 (14)	2.763 (15)	2.779 (15)	2.639 (15)	<0.001
	Subtotal amount	14.096 (88)	13.258 (82)	14.158 (80)	15.062 (78)	14.728 (74)	15.457 (75)	18.339 (76)	18.134 (76)	18.892 (75)	18.043 (72)	18.111 (72)	17.417 (72)	<0.001
Hospitals	Penicillin	0.088 (34)	0.184 (34)	0.296 (35)	0.410 (35)	0.415 (34)	0.409 (29)	0.476 (28)	0.511 (28)	0.572 (28))	0.596 (26)	0.649 (27)	0.702 (29)	<0.001
	Cephem	0.078 (30)	0.162 (30)	0.262 (31)	0.367 (32)	0.392 (32)	0.473 (34)	0.615 (36)	0.660 (36)	0.761 (37)	0.877 (38)	0.922 (38)	0.923 (38)	<0.001
	Fluoroquinolone	0.025 (9)	0.063 (11)	0.094 (11)	0.118 (10)	0.126 (10)	0.153 (11)	0.177 (10)	0.190 (10)	0.204 (10)	0.222 (9)	0.245 (10)	0.241 (9)	<0.001
	Macrolide	0.018 (7)	0.045 (8)	0.062 (7)	0.087 (7)	0.106 (8)	0.123 (8)	0.161 (9)	0.192 (10)	0.227 (9)	0.272 (12)	0.278 (11)	0.255 (10)	<0.001
	Subtotal amount	0.252 (1)	0.540 (3)	0.844 (4)	1.144 (5)	1.218 (6)	1.382 (6)	1.692 (7)	1.819 (7)	2.040 (8)	2.256 (9)	2.390 (9)	2.417 (9)	<0.001
General hospitals	Penicillin	0.313 (19)	0.426 (18)	0.494 (19)	0.552 (18)	0.653 (18)	0.569 (16)	0.611 (16)	0.627 (17)	0.636 (15)	0.643 (15)	0.648 (15)	0.635 (15)	0.001
	Cephem	0.431 (27)	0.671 (29)	0.796 (31)	0.953 (32)	1.438 (39)	1.174 (34)	1.272 (34)	1.221 (33)	1.345 (33)	1.434 (33)	1.432 (33)	1.443 (34)	<0.001
	Fluoroquinolone	0.265 (16)	0.336 (12)	0.350 (13)	0.403 (13)	0.424 (11)	0.470 (13)	0.553 (13)	0.598 (16)	0.675 (16)	0.745 (17)	0.758 (17)	0.767 (18)	<0.001
	Macrolide	0.185 (11)	0.303 (13)	0.346 (13)	0.395 (13)	0.436 (12)	0.465 (13)	0.488 (14)	0.489 (13)	0.532 (13)	0.582 (13)	0.515 (12)	0.488 (11)	<0.001
	Subtotal amount	1.593 (9)	2.280 (14)	2.564 (14)	2.934 (15)	3.614 (18)	3.403 (16)	3.727 (15)	3.679 (15)	3.982 (15)	4.258 (17)	4.225 (16)	4.228 (17)	<0.001
Long-term care hospitals	Penicillin	0.000	0.001 (20)	0.004 (28)	0.010 (29)	0.017 (26)	0.026 (22)	0.017 (23)	0.020 (26)	0.022 (25)	0.020 (24)	0.020 (23)	0.018 (20)	0.003
	Cephem	0.000	0.002 (40)	0.004 (28)	0.011 (32)	0.021 (32)	0.041 (32)	0.028 (39)	0.028 (36)	0.031 (36)	0.031 (37)	0.031 (36)	0.032 (36)	0.001
	Fluoroquinolone	0.000	0.001 (20)	0.002 (14)	0.005 (14)	0.008 (12)	0.018 (15)	0.008 (11)	0.008 (10)	0.011 (12)	0.011 (13)	0.014 (16)	0.015 (17)	0.002
	Macrolide	0.000	0.000	0.001 (7)	0.002 (5)	0.004 (6)	0.004 (3)	0.003 (4)	0.004 (5)	0.004 (4)	0.004 (4)	0.003 (3)	0.004 (4)	0.004
	Subtotal amount	0.001 (0)	0.005 (0)	0.014 (0)	0.034 (0)	0.065 (0)	0.117 (0)	0.071 (0)	0.076 (0)	0.086 (0)	0.083 (0)	0.086 (0)	0.087 (0)	<0.001
Total amount		15.943	16.142	17.656	19.272	19.753	20.482	23.958	23.836	25.107	24.732	24.899	24.219	<0.001

### Antimicrobial prescriptions by sociodemographic features

**Figure 3:**
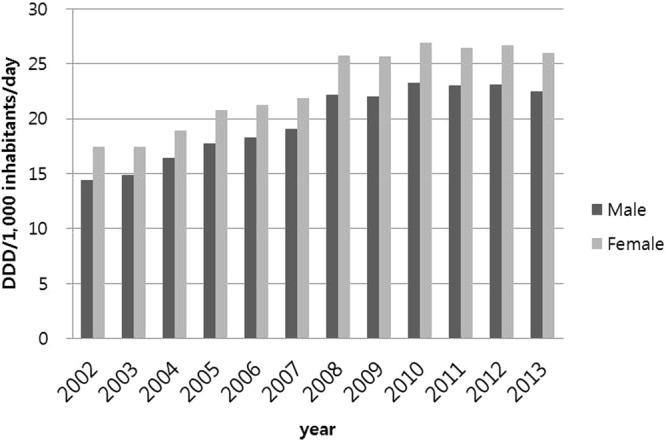
Antimicrobial prescription according to sex from 2002 to 2013.

**Figure 4:**
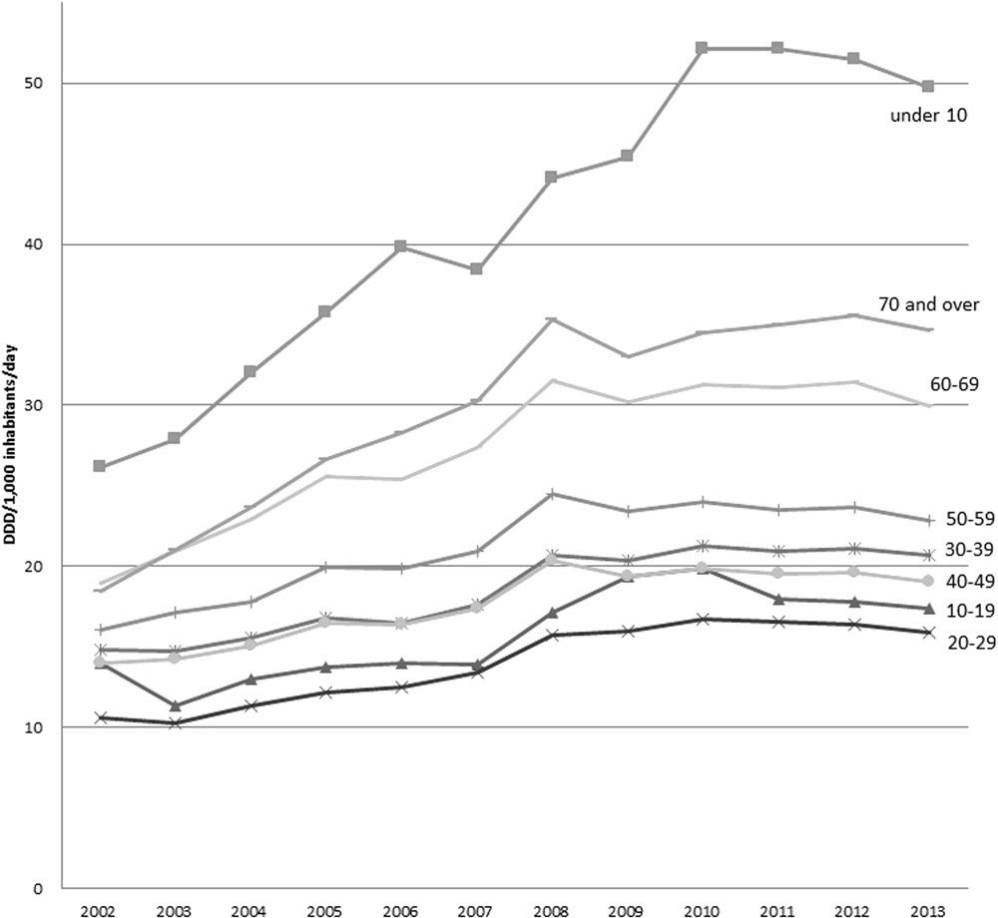
Antimicrobial prescription according to age group from 2002 to 2013.

No difference was observed in the use of antimicrobials according to gender (Fig. 3, *P* = 0.54), with antimicrobial prescription increasing every year in both males and females (Fig. 3, *P* < 0.001). The use of antimicrobials varied with age group (Fig. 4, *P* < 0.001). Antimicrobials were most frequently prescribed to children younger than 10 years, followed by adults aged 70 years or older and those aged 60–69 years (Fig. 4). In 2013, penicillins were the most frequently prescribed antimicrobial in children younger than 10 years, followed by cephems and macrolides, whereas cephems were the most frequently prescribed antimicrobial in adults aged 70 years or older, followed by fluoroquinolones, penicillins, and macrolides (data not shown).

**Figure 5:**
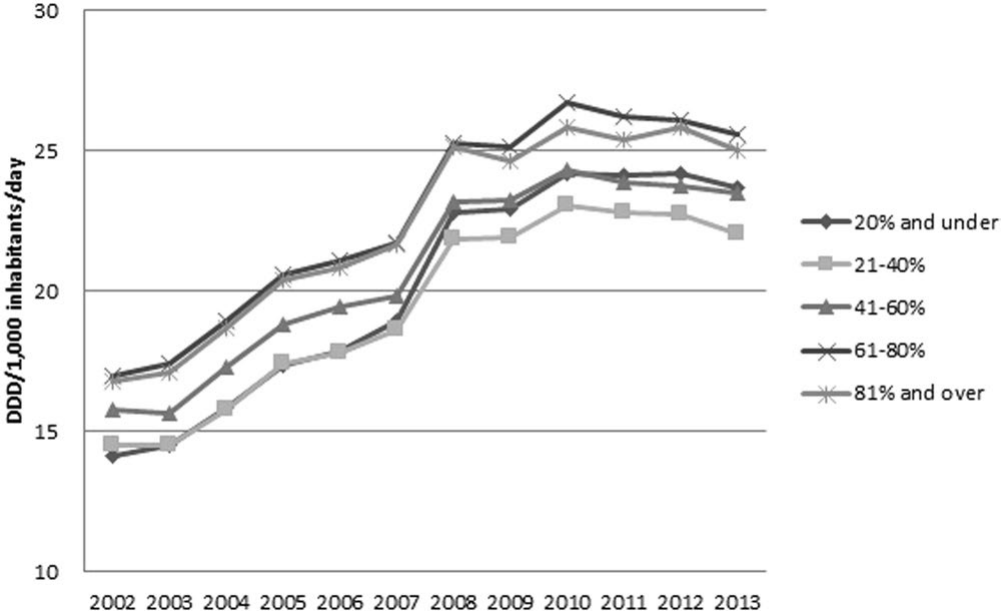
Antimicrobial prescription according to income levels of the sample into five groups (from upper 20 percentiles to lower 20 percentiles) from 2002 to 2013.

When antimicrobial prescription patterns were analyzed by stratifying the income levels of the sample into five groups (from the upper 20th percentile to the lower 20th percentile), no difference was found according to income level (Fig. 5, *P* = 0.65), with antimicrobial prescriptions increasing every year in all five income groups (Fig. 5, *P* < 0.001). Areas of residence were classified into 17 cities and provinces using the subjects’ addresses on resident registrations to examine differences in antimicrobial prescription patterns across regions. Antimicrobial prescriptions increased in all cities and provinces (*P* < 0.001), without regional differences (*P* = 0.47, data not shown).

### Antimicrobial prescriptions by major illness

**Figure 6:**
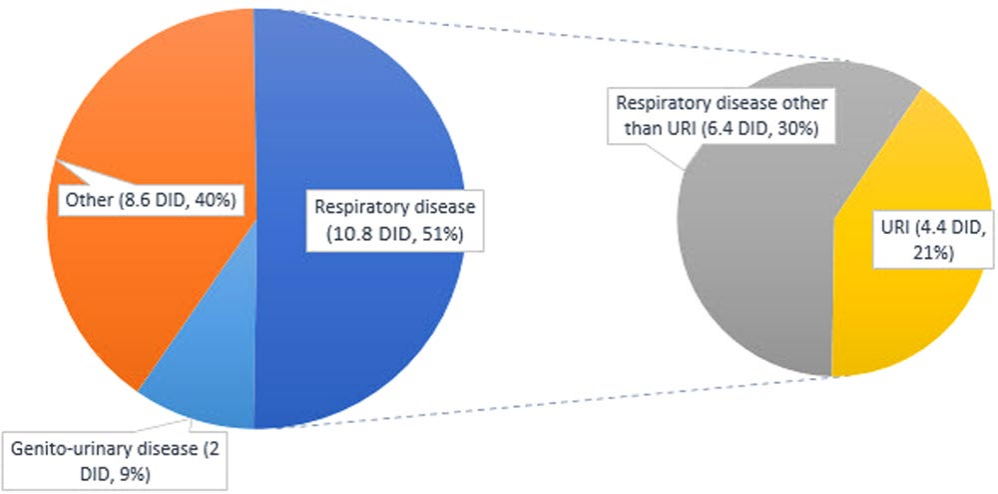
Average antimicrobial prescription according to major illness from 2002 to 2013 (DID: Daily Defined Dose/1,000 inhabitants/days; URI: upper respiratory tract infection).

For major illness prescribed for antimicrobials, more than half of all antimicrobial agents used were due to respiratory diseases (Fig. 6). Antimicrobial prescriptions for respiratory diseases increased every year (Table 3, *P* < 0.001). In 2013, 48% (11.683 DDD/1,000 inhabitants/day) of total antibiotic usage was due to respiratory diseases. Although not statistically significant, antimicrobial prescriptions for URIs decreased from 4.9 DDD/1,000 inhabitants/day in 2002 to 3.625 in 2013 (*P* = 0.109, Table 3). Antimicrobial prescriptions for genitourinary diseases, including urinary tract infections, increased every year (Table 3, *P* < 0.001).

**Table 3:** Antimicrobial prescription according to illness (DDD/1,000 inhabitants/days).

Illness	Class	2002	2003	2004	2005	2006	2007	2008	2009	2010	2011	2012	2013	*P*
Respiratory disease	Cephem	1.919 (21)	1.813 (21)	1.868 (20)	2.059 (20)	2.216 (22)	2.308 (23)	3.042 (24)	3.270 (26)	3.391 (26)	3.105 (25)	3.234 (26)	3.113 (26)	<0.001
	Macrolide	0.686 (7)	0.838 (10)	0.952 (10)	1.150 (11)	1.305 (13)	1.557 (15)	1.973 (16)	2.167 (17)	2.418 (18)	2.559 (21)	2.498 (20)	2.307 (19)	<0.001
	Penicillin	4.922 (54)	4.431 (53)	5.125 (56)	5.651 (56)	5.232 (53)	5.030 (50)	5.895 (48)	5.755 (46)	5.835 (45)	5.345 (44)	5.441 (44)	5.281 (45)	0.075
	Fluoroquinolone	0.519 (5)	0.503 (6)	0.498 (5)	0.559 (5)	0.543 (5)	0.604 (6)	0.764 (6)	0.745 (6)	0.700 (5)	0.694 (5)	0.737 (6)	0.697 (5)	<0.001
	Subtotal amount	9.002 (56)	8.265 (51)	9.032 (51)	9.988 (51)	9.777 (49)	9.968 (48)	12.171 (50)	12.390 (51)	12.759 (50)	12.074 (48)	12.232 (49)	11.683 (48)	<0.001
Upper respiratory tract infection	Cephem	1.012 (20)	0.928 (21)	0.934 (20)	1.028 (20)	0.855 (21)	0.935 (23)	1.229 (24)	1.269 (26)	1.289 (26)	1.045 (26)	1.029 (26)	0.963 (26)	0.264
	Macrolide	0.266 (5)	0.269 (6)	0.297 (6)	0.367 (7)	0.334 (8)	0.394 (9)	0.529 (10)	0.566 (11)	0.644 (13)	0.558 (13)	0.557 (14)	0.504 (13)	<0.001
	Penicillin	2.879 (58)	2.520 (58)	2.839 (62)	3.106 (62)	2.438 (60)	2.316 (57)	2.776 (56)	2.603 (54)	2.562 (53)	2.128 (53)	2.054 (52)	1.951 (53)	0.005
	Fluoroquinolone	0.243 (4)	0.226 (5)	0.208 (4)	0.225 (4)	0.189 (4)	0.198 (4)	0.241 (4)	0.225 (4)	0.193 (4)	0.160 (4)	0.156 (4)	0.132 (3)	0.003
	Subtotal amount	4.900 (30)	4.279 (26)	4.552 (25)	4.982 (25)	4.000 (20)	4.012 (19)	4.949 (20)	4.817 (20)	4.824 (19)	3.999 (16)	3.886 (15)	3.625 (14)	0.109
Genito-urinary disease	Cephem	0.173 (12)	0.233 (14)	0.258 (16)	0.297 (18)	0.330 (19)	0.388 (19)	0.460 (19)	0.453 (19)	0.471 (20)	0.479 (20)	0.484 (20)	0.488 (21)	<0.001
	Macrolide	0.024 (1)	0.029 (1)	0.028 (1)	0.034 (2)	0.048 (2)	0.052 (2)	0.061 (2)	0.061 (2)	0.067 (2)	0.070 (2)	0.077 (3)	0.074 (3)	<0.001
	Penicillin	0.382 (27)	0.381 (23)	0.361 (22)	0.354 (21)	0.327 (18)	0.255 (12)	0.229 (9)	0.203 (8)	0.192 (8)	0.185 (7)	0.172 (7)	0.167 (7)	<0.001
	Fluoroquinolone	0.352 (25)	0.482 (30)	0.502 (31)	0.532 (32)	0.566 (32)	0.668 (34)	0.829 (34)	0.814 (35)	0.829 (35)	0.847 (35)	0.843 (35)	0.796 (34)	<0.001
	Subtotal amount	1.381 (8)	1.593 (9)	1.589 (8)	1.646 (8)	1.724 (8)	1.979 (9)	2.372 (9)	2.322 (9)	2.341 (9)	2.383 (9)	2.382 (9)	2.302 (9)	<0.001
Urinary tract infection	Cephem	0.057 (10)	0.073 (11)	0.078 (12)	0.088 (13)	0.096 (13)	0.114 (14)	0.138 (14)	0.140 (15)	0.152 (16)	0.157 (16)	0.170 (17)	0.182 (19)	<0.001
	Macrolide	0.015 (2)	0.015 (2)	0.015 (2)	0.018 (2)	0.021 (3)	0.022 (2)	0.023 (2)	0.022 (2)	0.025 (2)	0.023 (2)	0.026 (2)	0.025 (2)	<0.001
	Penicillin	0.080 (14)	0.093 (14)	0.089 (13)	0.086 (12)	0.084 (12)	0.076 (9)	0.072 (7)	0.064 (7)	0.065 (6)	0.065 (6)	0.063 (6)	0.061 (6)	<0.001
	Fluoroquinolone	0.177 (32)	0.245 (37)	0.248 (38)	0.272 (40)	0.282 (40)	0.333 (41)	0.404 (43)	0.388 (43)	0.411 (43)	0.426 (45)	0.434 (44)	0.412 (43)	<0.001
	Subtotal amount	0.542 (3)	0.656 (4)	0.641 (3)	0.671 (3)	0.700 (3)	0.801 (3)	0.923 (3)	0.892 (3)	0.938 (3)	0.940 (3)	0.968 (3)	0.942 (3)	<0.001
Total amount		15.943	16.142	17.656	19.272	19.753	20.482	23.958	23.836	25.107	24.732	24.899	24.219	<0.001

All classes of antimicrobial were included in total amount. (% of subtotal DDD or % of total DDD).

By class, penicillins were the most frequently prescribed antimicrobial for respiratory diseases (5.281 DDD/1,000 inhabitants/day) in 2013, followed by cephems (3.113), macrolides (2.307), and fluoroquinolones (0.697), and similar trend was observed for URIs. For genitourinary diseases, fluoroquinolones were the most frequently prescribed antibiotics (0.796), followed by cephems (0.488), and a similar trend was observed for urinary tract infections.

When antibiotic use was compared between 2013 and 2002, cephem, macrolide and fluoroquinolone use increased, but penicillin use was not increased for respiratory diseases. For genitourinary diseases, prescription of cephem, macrolide, penicillin and fluoroquinolone increased (see Table 3).

## Discussion

This study explored the changes of antimicrobial usage in Korea from 2002 to 2013 through analyzing the NHIS-NSC data using DDD developed by the WHO for drug statistics methodology.

Antimicrobial resistance in human pathogens shows substantial geographic differences across the globe. Although antimicrobial resistance remains low in Northern European countries, it is high in Southern European, Latin American and Asian countries. In Asia, extended spectrum β-lactamase (ESBL)-producing Enterobacteriaceae are of concern and are increasing. In 2009 and 2010, 28 percent of Enterobacteriaceae isolated from the patients with urinary tract infections in 11 countries were ESBL producers^18^. In China, in 2011, ESBL-producing E. coli accounted for 71 percent of E. coli isolates, and more than half of K. pneumoniae strains produced ESBL^19^. Infections with carbapenem-resistant Enterobacteriaceae are increasingly reported from healthcare facilities, not only in developed countries^20^, but also in low- and middle income countries. Treatment for carbapenem-resistant Enterobacteriaceae is much more complicated than ESBL-producing stains because of limited treatment options.

Between 2000 and 2010, global consumption of antibiotic drugs increased by 36%^21^. There is a high variability of antimicrobial consumption across countries. Among the Organisation for Economic Co-operation and Development (OECD) countries, Chile and Netherlands reported the lowest volume (9.4 and 11.4 DDD/1,000 inhabitants/day, respectively) and Greece and Italy reported 30.1 and 34.9 DDD/1,000 inhabitants/day, respectively in 2013^22^. Antimicrobial use in Japan was 14.7 in 2009 and 15.8 DDD/1,000 inhabitants/day^23^. Korea remains a country with heavy antimicrobial usage^16,24^; its 2013 national average (28.4 DDD/1,000 inhabitants/day) exceeds the OECD average (20.4 DDD/1,000 inhabitants/day)^22^. New systems were introduced in Korea upon the turn of the 21^st^ century to increase the quality of medicine and reduce the use of antimicrobials^25,26^, including the separation of dispensing and prescribing in 2000, drug-use assessment (including antimicrobials) in 2001, public reporting of the rate of antibiotics use for URI in 2006, and assessment of antimicrobial use for surgical prophylaxis^27^.

The changing patterns of antibiotics usage in Korea have been reported from the past^28^. A study that compared antimicrobial usage in Korea before and after the separation of dispensing and prescribing in July 2000 reported that antimicrobial usage before the separation was 28.1 DDD/1,000 inhabitants/day in 1998 and 30.8 in 1999, which decreased to 19.8 in 2001 and 17 in 2002 after the separation^25^. Studies that analyzed antimicrobial usage after the separation of dispensing and prescribing drugs using health insurance found that usage was 23.62 in 2004^11^. A previous study, ‘Annual Products of Medicine in 2008′, analyzed the cost and amounts of antibiotics produced and compared them with previous data. The cost and amounts of antibiotics are indirect parameters of drug utilization, and that study did not consider imported antibiotics. In this study, the DDD was used because it facilitates the presentation and comparison of drug consumption statistics at international, national and regional levels. Although drug consumption can be measured in cost, number of units, number of prescriptions or the physical quantity of drugs, these variables can vary between regions and countries over time. Because of such limitation in comparisons of drug consumption at an international level, a technical unit of measurement, DDD, is recommended by the WHO to measure drug utilization in countries^29^. Moreover, the NHIS-NSC data used in this study were claim data that could be used to estimate nationwide antibiotics usage directly^14^.

This study found that antimicrobial usage increased, from 15.943 DDD/1,000 inhabitants/day in 2002 to 24.219 in 2013. Analysis of usage by year showed a sharp increase from 2002 to 2010, but had been consistent since 2010. Since antimicrobial usage is increasing despite the enforcement of relevant policies, it can be suspected that these policies are not effective enough, or that the growth of the elderly population has increased antimicrobial usage. However, the rate at which antibiotics use increases is declining in recent years, and it seems that the antibiotic policy is beginning to show its effects.

URI, a disease that encompasses simple colds, allergic rhinitis, middle ear infections, and pharyngitis, is viral in most cases, and antimicrobials are rarely required, suggesting that antimicrobial usage for this particular disease should be managed closely^30^. Although changing prescribing behaviors can be difficult, guidelines exist for acute rhinosinusitis^31^, acute uncomplicated bronchitis^32^, and pharyngitis^33^, and proven evidence-based methods can be used to optimize antibiotic therapy in outpatient settings^34–36^.

Over the last decade the Korean government has implemented a series of healthcare policies directed to URI consisting of drug utilization review in the outpatient service in 2001 and public reporting of the rate of antibiotics use for URI in 2006. As a result of government policy for URI, antimicrobial usage for URIs tended to decline, though the trend was not statistically significant, from 4.9 DDD/1,000 inhabitants/day in 2002 to 3.625 in 2013. The proportion of total antimicrobial usage for acute URIs decreased from 30% in 2002 to 14% in 2013. In particular, antimicrobials prescribed for URI decreased after public reporting of the rate of antibiotics use for URI in 2006 (4.982 in 2005 and 4.000 in 2006).

Perception change of primary physician and a public campaign for ‘appropriate antibiotic use’ could contribute to decrease of antimicrobial usage. Studies using clinical scenarios involving the common cold or standard patients showed that physicians’ responses in favor of antibiotic prescription in acute URTIs fell from 75.0% in 1991 to 54.7% in 2003 and to 27.2% in 2010 although the clinical cases and study subjects differed between the studies^37–40^. A public campaign for ‘appropriate antibiotic use’ organized by the Korean Society of Infectious Diseases and the Korean Society for Chemotherapy, and sponsored by the Korea CDC, was launched in Korea in 2011^41^.

In 2008, the use of antimicrobials increased significantly. After the implementation of public reporting in 2006, diagnostic coding which has not been designated for antibiotic use evaluation were increased significantly. For example, the most common respiratory tract infections was J03 acute tonsillitis in 2005 but changed to J20 acute bronchitis in 2009. Increased use of antimicrobials in 2008 may be explained in part by diagnostic shift in the outpatient setting although the cause of increasing trends in total antimicrobial usage needs to be determined^27^.

The use of aminoglycosides decreased from 1.494 in 2002 (10% of total antimicrobial usage) to 0.807 in 2013 (3% of total antimicrobial usage). Although amoxicillin was the most popular penicillin in 2002, amoxicillin-clavulanate was more popular in 2013, suggesting a preference for combined drugs to overcome bacterial resistance. Whereas the use of penicillins did not increase, the use of cephems increased more than two-fold over our study period. Cephem use was only half of that of penicillins in 2002, but prescriptions increased to a level comparable with that of penicillins in 2013. The most frequently used cephem was cefaclor; its usage in 2013 was 3.222 DDD/1000 inhabitants/day, which accounted for half of the total cephem usage (7.211). The fact that the use of cefaclor, a second-generation cephem, exceeded that of oral third-generation cephems such as cefixime and cefpodoxime (though their use increased as well) could be related to insurance policies that do not cover primary treatment with third-generation cephems. Furthermore, the side effects (e.g., diarrhea) of amoxicillin-clavulanate, which is used to overcome resistance, could contribute to the preference for cefaclor. Macrolide and fluoroquinolone prescriptions also increased, and prescriptions for clarithromycin, levofloxacin, and ciprofloxacin increased by particularly high degrees. Use of sulfamethoxazole-trimethoprim, a drug typically used to treat urinary tract infections, declined, possibly due to concerns of resistance of *Escherichia coli*—the most common pathogen in urinary tract infections.

Regarding age, antimicrobials were most heavily used in children under the age of 10, followed by adults aged 70 years or older and those aged 60–69 years. Penicillins were the most popular antimicrobial among children. Fluoroquinolones are not used in children due to the potential side effects. Cephems were preferred over penicillins among adults aged 20 years or older, and cephems and fluoroquinolones were preferred over penicillins among adults aged 70 years or older in 2013.

In this study analyzed all antimicrobial used, there was no difference was found according to income level. Recent study for antimicrobial used for acute URIs found that income level was negatively correlated with prescription rate and suggested demand factors as well as supply factors contributed antibiotic overuse^15^. We hypothesize that demand factors have a greater impact on mild illnesses such as acute URIs.

In 2013, 72% of total prescriptions occurred in clinics. This is attributable to the fact that a greater number of patients are treated in clinics than in other types of medical institutions. Thus, antimicrobial usage in clinics should be closely monitored.

In conclusion, antimicrobial usage increased in Korea from 2002 to 2013, with 70% of the prescriptions made in clinics, and penicillins and cephems being the most popular classes of antimicrobials. In 2013, 48% of all antimicrobials were prescribed for respiratory diseases, and antimicrobial prescriptions for URIs are declining. On the cusp of a post-antibiotics era, monitoring antimicrobial use can be an important strategy to prolong the activities of existing antimicrobials.

### Data availability

The datasets generated during the current study are available from the corresponding author on reasonable request.
